# The Association between Physical Activity During the Day and Long-Term Memory Stability

**DOI:** 10.1038/srep38148

**Published:** 2016-12-02

**Authors:** Matthew B. Pontifex, Kathryn L. Gwizdala, Andrew C. Parks, Karin A. Pfeiffer, Kimberly M. Fenn

**Affiliations:** 1 Michigan State University, 308 W. Circle Dr, East Lansing, MI 48824, United States.

## Abstract

Despite positive associations between chronic physical activity and memory; we have little understanding of how best to incorporate physical activity during the day to facilitate the consolidation of information into memory, nor even how time spent physically active during the day relates to memory processes. The purpose of this investigation was to examine the relation between physical activity during the day and long-term memory. Ninety-two young adults learned a list of paired-associate items and were tested on the items after a 12-hour interval during which heart rate was recorded continuously. Although the percentage of time spent active during the day was unrelated to memory, two critical physical activity periods were identified as relating to the maintenance of long-term memory. Engaging in physical activity during the period 1 to 2-hours following the encoding of information was observed to be detrimental to the maintenance of information in long-term memory. In contrast, physical activity during the period 1-hour prior to memory retrieval was associated with superior memory performance, likely due to enhanced retrieval processing. These findings provide initial evidence to suggest that long-term memory may be enhanced by more carefully attending to the relative timing of physical activity incorporated during the day.

One aspect of cognition that appears particularly sensitive to chronic levels of physical activity (i.e., the habitual expenditure of energy above and beyond the normal physiological demands of the day) and aerobic fitness (i.e., the attribute characterizing the ability to sustain aerobic physical activities) is long-term memory. However, in a critical misappropriation of the literature, such evidence has increasingly been used to argue for the incorporation of physical activity opportunities during the school day. While such bouts of physical activity may eventually amass to create more long-term changes in memory, we have little understanding of how best to incorporate physical activity during the day to facilitate the consolidation of information into memory nor even how time spent physically active during the day relates to memory processes. Accordingly, the aim of the present investigation was to examine the relation between physical activity during the day and the maintenance of information within long-term memory. This aim was first investigated relative to the total accumulated physical activity during the day – in line with the approach of current physical activity recommendations^1^. Conversely however, physical activity is typically engaged in through bouts of activity; thus, the second aim of this investigation was to determine if there may be critical periods during the day when physical activity engagement was more strongly associated with long-term memory.

The ability to store declarative or explicit information such as facts or events for long periods of time has been found to be dependent upon a network of structures such as the hippocampus and entorhinal cortex in the medial temporal lobe^2^, which appear sensitive to chronic levels of physical activity. A sizable body of support for such assertions are drawn from rodent models, where wheel-running has been found to result in enhanced hippocampal-dependent learning and memory^3^ and neurogenesis in the dentate gyrus of the hippocampus^4^. This body of literature collectively suggests that chronic physical activity engagement and the associated attribute of aerobic fitness appear to enhance neural structures that facilitate the storage of declarative information in long-term memory. With regard to human models however, although a meta-analytic review conducted by Smith and colleagues^5^, observed a modest relationship (Hedges’s g = 0.128) between chronic aerobic exercise and declarative memory in older adults; other meta-analytic reviews have concluded that the wide variation in study designs and memory assessments make such gross generalizations difficult to support^6^. Although the vast majority of research in this area has focused on older adults where the effects of chronic physical activity may be more readily apparent due to age-related degradation of memory; evidence for a relationship between chronic physical activity and memory has also been observed for younger populations. Indeed, given the growing epidemic of sedentary behaviors among industrialized societies^1^, younger populations are engaging in physical activity to a lesser extent. Furthermore, recent evidence suggests that lower-fit college-aged adults exhibit poorer declarative memory retention than their higher-fit peers^7^. Even among school-aged children, longitudinal physical activity interventions have been shown to enhance hippocampal-dependent relational memory^8^.

However, the consolidation of declarative memory (i.e., the process by which information is strengthened and made more resistant to interference or decay^9^); has also been demonstrated to be influenced by behavioral actions and physiological state between memory encoding and retrieval^10^,^11^,^12^,^13^,^14^,^15^,^16^. The investigation of physical activity during this period however, has received surprisingly little attention with the vast majority of research instead focusing on the effects of sleep^12^,^13^,^14^,^17^. While some insight is provided by investigations focusing on the effects of a single short duration bout of physical activity on long term memory, such investigations have taken a programmatically different approach to assessing long-term memory. Specifically, the sleep literature assesses the stability of long-term memory over a period of 12 to 24 hours, with memory encoding occurring prior to sleep and memory retrieval occurring following sleep^13^. In contrast, investigations of acute exercise allow substantially less time for memory decay to occur with encoding and retrieval separated by a period of only 12 minutes in order to allow for the assessment of memory retrieval prior to and again following exercise^18^,^19^. Thus, it is unsurprising that such investigations have failed to observe exercise-related effects for the total number of memory items recalled following approximately 40 minutes of moderate intensity physical activity^18^,^19^. It should be noted, however, that although these investigations utilized a strong-empirical approach incorporating a within-subject, repeated measures design, a key limitation as it applies to long-term memory is the potential for interference to be induced as multiple word lists are encoded (i.e., retroactive inhibition where memory consolidation is impaired as a result of similarity between word lists^20^). Indeed, when focusing only on the initial (primacy) and final (recency) portions of the memory items where such interference effects should be mitigated, exercise-related enhancements in memory were observed^18^,^19^. Interestingly, although Labban and Etnier^21^ observed a lack of modulations in long-term memory when exercise was performed during a 35 minute interval between encoding and retrieval (see also^22^), superior memory performance was observed when participants exercised for 30 minutes at a moderate intensity prior to memory encoding. Indeed, converging evidence suggests that physical activity prior to the encoding of information is beneficial for long-term memory^21^,^23^,^24^,^25^. However, the extent to which engaging in physical activity during the period between encoding and retrieval is less well understood. Thus, prior to the recommendation for public health policies that attempt to incorporate physical activity during the day, a greater understanding of the relative timing of the exercise as it relates to long-term memory is necessary as there may be critical periods during which physical activity is detrimental to the maintenance of information within long-term memory.

Accordingly, the aim of this investigation was to examine the relation between physical activity during the day and the maintenance of information within long-term memory. Replicating the empirical approach utilized within the sleep literature, memory consolidation was assessed across a 12 hour interval using a between-subjects design to reduce the potential for confounds related to interference, while at the same time controlling for variation in performance relating to aerobic fitness. Given such a large retention interval and the lack of understanding in the literature of the relative time-course of the effects of physical activity on long-term memory, this investigation first characterized the relation between the maintenance of information in long-term memory and time spent in sedentary behavior and moderate to vigorous physical activity during the period between memory encoding and retrieval. It was hypothesized that spending more time in moderate to vigorous physical activity would relate to superior maintenance of information in long term memory. To determine if physical activity engagement more strongly relates to long-term memory during certain critical periods, exploratory analysis were conducted to characterize the relationship between long-term memory and time spent in sedentary behavior and moderate to vigorous physical activity during a sliding 20 minute period; first to assess for critical periods following the encoding of information into long-term memory and then for critical periods prior to the retrieval of information. Accordingly, the contribution of this investigation was in providing insight into the relation between physical activity during the day and long-term memory.

## Method

### Participants

Analyses were conducted on a sample of 92 undergraduate students recruited from Michigan State University. All experimental protocols were approved by the Michigan State University Institutional Review Board and all methods were carried out in accordance with those protocols and relevant guidelines and regulations regarding the use of human subjects. Participants provided written informed consent, completed a health history and demographics questionnaire, reported being free of any neurological diseases or physical disabilities, and indicated normal or corrected-to-normal vision. Of the 151 participants initially recruited, 28 participants were excluded for failing to complete the experiment, 15 participants were excluded for having less than 9.5 hours of heart rate data recorded, 10 participants were excluded for failing to complete the test of aerobic fitness, and 4 participants were excluded for obtaining less than 5 hours of sleep on the night prior to the experiment—to avoid potential confounds related to sleep deprivation^26^. Demographic and fitness data for all participants are provided in Table 1.

### Paired Associates Memory Task

Long-term memory was assessed using a paired associates memory task over the course of two sessions, separated by 12 hours. During the first session, participants studied 68 semantically related word pairs^7^,^13^. Word pairs were randomly displayed (1.3° vertical visual angle) at the center of the screen. Each pair was presented for 3500 ms with a 1000 ms inter-stimulus interval (ITI). Immediately after training, participants were given a cued recall test on 60 word pairs. The first and final four pairs were not presented during the test, to control for primacy and recency effects on memory performance. During the test, the first word of each pair was presented at the center of the computer screen, and participants were asked to type the second word in a box directly below the first word of the pair. There was no time limit to respond. After each response, participants were first told whether their response was correct or incorrect and were then shown the correct word pair, regardless of response. Words were presented randomly during study and test. Participants were trained to a criterion of 33% correct. After criterion was achieved, participants were given a final cued-recall test without feedback. During the second session (approximately 12 hours following the first session), participants were again given a cued recall test on 60 word pairs, without feedback^7^. As performance on the paired associates memory task during the second session was highly correlated with performance during the first session (r = 0.9, p < 0.001), long-term memory performance was quantified by calculating the number of paired-associate items that were maintained across the 12-hour retention interval, corrected by the total number of items recalled in the final test during session 1 ([items maintained/total items recalled in session 1] × 100). Thus, higher values indicated better memory retention. This approach is modeled after approaches previously employed in the literature^7^,^12^,^13^,^14^ and ensured that any observed effects could not be explained by greater maintenance for individuals who demonstrated poorer performance during session 1.

### Aerobic Fitness Assessment

Aerobic fitness was assessed using a test of maximal oxygen consumption (VO_2_max), which describes the physiological limit to the rate at which an individual can deliver/consume oxygen^7^,^27^. Relative peak oxygen consumption (ml/kg/min) was measured using a computerized indirect calorimetry system (ParvoMedics True Max 2400) while participants ran or walked on a motor-driven treadmill at a constant speed with incremental increases of 2.5% grade every two minutes until the participant was no longer able to maintain the exercise intensity^27^. Maximal effort was evidenced by attainment of a plateau in oxygen consumption corresponding to an increase of less than 2 ml/kg/min despite an increase in workload. Participants who did not achieve criterion for maximal effort all achieved peak effort as evidenced by attainment of at least two of the following three criteria: (1) a peak heart rate within 10 beats per minute of age-predicted maximum (i.e., 220-age); (2) respiratory exchange ratio ≥ 1.1; or (3) OMNI perceived exertion scale rating >7^28^. Aerobic fitness percentiles were extracted from normative data provided by Shvartz and Reibold^29^.

### Procedure

**Figure 1:**
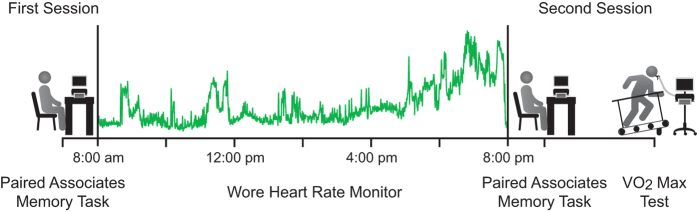
Schematic illustration of the progression of procedures utilized by the current investigation. Participants first completed the training and initial assessment of the paired associates memory task. Participants then wore a heart rate monitor that continuously recorded heart rate in 20 second epochs throughout the day. Approximately 12 hours later, participants were tested on the paired associates memory task and then completed a brief test of their aerobic fitness.

Participants were asked to come into the laboratory on two occasions separated by an approximately 12 hour period (mean: 11:34:20 ± 00:19:13). During the first session, participants completed the training and initial assessment of the paired associates memory task. On the second session, participants were tested on the paired associates memory task and then were asked to complete a brief test of their cardiorespiratory fitness (see Fig. 1). During the period separating the two sessions, participants were asked to wear a Polar RCX3 heart rate monitor (Polar Electro, Finland) that continuously recorded heart rate (HR) in 20 second epochs. To facilitate comparison between individuals, each heart rate epoch was converted from heart rate in beats per minute to the percent of heart rate reserve (i.e., the difference between the maximum heart rate and minimum heart rate) that heart rate corresponded to^27^. For example, a heart rate of 90 bpm in an individual with a minimum heart rate of 75 and a maximum heart rate of 200 would correspond to 12% of heart rate reserve (HR_current_ − HR_min_)/(HR_max_ − HR_min_). Maximum heart rate was quantified as the maximum heart rate achieved during the test of cardiorespiratory fitness, while minimum heart rate was quantified as the lowest mean heart rate recorded over a 10 minute window during the approximately 12 hour period between sessions. Physical activity intensity was established using cutpoints provided by the American College of Sports Medicine^27^, with sedentary behavior quantified as (<30% HRR, Mean HR: 83.4 ± 9.0 bpm), light physical activity quantified as (30 to 39.9% HRR, Mean HR: 113.3 ± 7.2 bpm), moderate physical activity quantified as (40 to 60% HRR, Mean HR: 129.1 ± 6.9 bpm), and vigorous physical activity quantified as (>60% HRR, Mean HR: 155.3 ± 9.0 bpm). Although physical activity is often assessed through the use of accelerometry based measures^30^ the use of heart rate in the present investigation allowed for the objective characterization of energy expenditure relative to each participant’s individual cardiac capacity thereby better accounting for individual differences in aerobic fitness. Participants were not specifically instructed to avoid or engage in physical activity during the 12 hour period, but were instructed to not nap or sleep during this period.

### Statistical Analysis

All data analyses were performed in PASW Statistics, 20.0 (IBM, Somers, NY) and Matlab R2014a (The Mathworks, Inc., MA) utilizing a family wise alpha level of *p* = 0.05. Prior to analysis, all study variables were screened for homoscedasticity and normality. Bivariate correlation analyses were then conducted using Spearman’s rank correlation coefficient between demographic factors and long-term memory. Stepwise hierarchical linear regression analyses were performed using a generalized linear regression approach to explain variance in long-term memory as related to aerobic fitness. This was undertaken by regressing long-term memory on descriptive factors (i.e., Age, Sex [0 = Female, 1 = Male], Race [0 = white, 1 = nonwhite], and Body Mass Index) that were statistically significant correlates in Step 1 to judge the independent contribution of aerobic fitness (as assessed using VO_2_max percentile) in Step 2 for explaining variance beyond that of the descriptive variables^7^.

Following this, the independent contribution of the proportion of time spent physically active during the day was assessed in Step 3 for explaining variance beyond that of descriptive variables and aerobic fitness. This analysis was performed by assessing the association between long-term memory and the percentage of time spent between sessions in sedentary behavior (<30% HRR) and and in a separate analysis, moderate-to-vigorous physical activity (≥40% HRR). Although time sepent in sedentary behavior and in moderate-to-vigorous physical activity are highly correlated (*r* = −0.92, *p* < 0.001) and mutually exclusive (i.e., in the present design a participant cannot be both sedentary and physically active at the same time point); they represent distinct components of the physical activity continuum seperated by light physical activity (30 to 39.9% HRR). Thus, seperately analyzing these two zones allows for differentiation of if memory is enhanced by simply avoiding sedentary behavior or if accumulated physical activity must be of a sufficient intensity to incur benefits.

**Figure 2:**
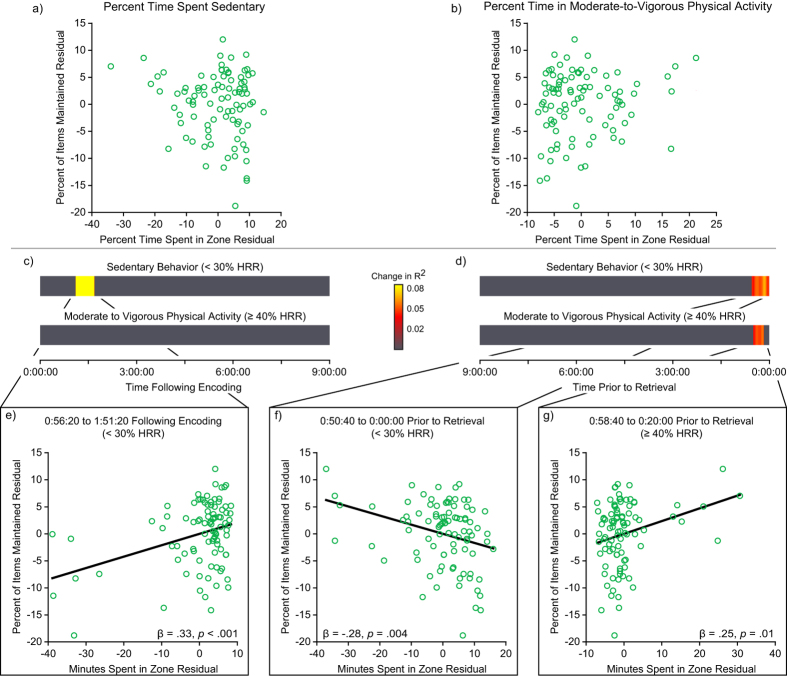
The plots along the top of the figure present the relationship between the proportion of items maintained between session 1 and session 2 and the percent time spent sedentary (**a**) and the percent time spent in moderate-to-vigorous physical activity (**b**) throughout the entire day, controlling for the influence of Age, Sex, and Aerobic Fitness. The middle plots present the change in R^2^ from the hierarchical linear regression for the 20 minute period surrounding each point across a 9 hour period time-locked to the first session (**c**) and a 9 hour period time-locked to the second session (**d**) controlling for the influence of Age, Sex, and Aerobic Fitness. The bottom plots provide scatterplots of the relationship between residuals for the proportion of items maintained between session 1 and session 2 and the number of minutes spent sedentary (**e**,**f**), and in moderate-to-vigorous physical activity (**g**) during the critical periods identified, controlling for the influence of Age, Sex, and Aerobic Fitness.

To determine the existence of any critical periods for physical activity, analyses were conducted to examine the independent association between long-term memory and bouts of sedentary behavior and moderate-to-vigorous physical activity. Rather than speculate as to the particular timing of such bouts of activity, a data-driven approach was utilized to characterize the relation between long-term memory and the minutes spent in each HR zone during 20 minute periods throughout the day. To account for variability in the time separating the first session from the second session (i.e., approximately 11.5 hours ± 19 minutes), we examined this relationship with the heart rate data time-locked to the first session to assess for any relationships occurring post-encoding; and then repeated this process with the heart rate data time-locked to the second session to assess for any relationships occurring prior to retrieval. For each of these instances, we utilized a generalized linear regression approach to characterize the relationship between long-term memory and the minutes spent in each HR zone, while controlling for the influence of descriptive variables and aerobic fitness. This process then incrementally performed hierarchical linear regression analysis of these relationships, with each iteration shifting this 20 minute period by one 20 second epoch over the span of a 9 hour period (i.e., 9 hours following the first session and then the process was repeated for the 9 hours prior to the second session; see Fig. 2).

Given the large number of analysis performed, a cluster-threshold multiple probability criterion was utilized; which is a common approach to control for the potential for Type I error in neuroimaging investigations that utilize a large number of statistical tests on related sampling points^31^,^32^. The conceptual justification for this approach is that spurious or false positive findings should exhibit less of a tendency to form clusters (i.e., multiple contiguous comparisons all at or below the specified alpha level) than true regions of significance, with larger cluster sizes relating to a decreased probability of a false positive finding. Thus, this approach conceptually maps onto the attempt to define critical periods relevant to long-term memory retention. The cluster-threshold multiple probability criterion required a cluster of 30 temporally contiguous comparisons (representing a total span of at least 30 minutes) at or below alpha = 0.05 for the comparison to be considered statistically significant. Once these periods were defined, hierarchical linear regression analyses were then performed to examine the relationship between long-term memory and the minutes spent in each HR zone during the critical periods identified, while controlling for the influence of descriptive variables and aerobic fitness. Overall means and variability measures are provided in Table 1. Statistical summaries of the correlational and regression analyses are provided in Tables 2 and 3, respectively.

## Results

### Performance on the Paired Associates Memory Task

Memory recall was observed to decrease over time, with poorer performance observed at session 2 (47.0 ± 7.8 words) relative to session 1 (49.4 ± 6.8 words; *t* (91) = 7.2, *p* < 0.001, *d* = 1.36).

### Aerobic Fitness

Hierarchical regression analysis indicated that individuals with greater-fitness exhibited more stable long-term memory as indexed by a greater proportion of items maintained between session 1 and session 2 (*β* = 0.225, *t* (88) = 2.2, *p* = 0.032), even after controlling for Age and Sex (see Table 3).

### Percentage of Time Spent in Heart Rate Zones during the Entire Day

No relationship to long-term memory was observed for either the percentage of time spent sedentary (*β* = −0.130, *t* (87) = 1.3, *p* = 0.19), or the percentage of time spent in moderate-to-vigorous physical activity (*β* = 0.119, *t* (87) = 1.2, *p* = 0.23), after controlling for Age, Sex, and Aerobic Fitness (see Fig. 2).

### Bouts of Activity during the Day

Hierarchical regression analysis across the 9-hour period time-locked to the first session (i.e., following encoding) — controlling for Age, Sex, and Aerobic Fitness — revealed one critical window for sedentary behavior related to long-term memory retention. Individuals who spent more time sedentary 0:56:20 to 1:51:20 following the first session exhibited greater long-term memory as indexed by a greater proportion of items maintained between session 1 and session 2 (*β* = 0.27, *t* (87) = 3.5, *p* < 0.001; see Fig. 2, Table 3).

Hierarchical regression analysis across the 9-hour period time-locked to the second session (i.e., prior to retrieval) — controlling for Age, Sex, and Aerobic Fitness — revealed one critical window for sedentary behavior and another for moderate-to-vigorous physical activity related to long-term memory retention. Individuals who spent less time sedentary 0:50:40 up to the start of the second session, (*β* = −0.28, *t* (87) = 2.9, *p* = 0.004), and more time in moderate-to-vigorous physical activity 0:58:40 to 0:20:00 prior to the second session, (*β* = 0.24, *t* (87) = 2.6, *p* = 0.012), exhibited greater long-term memory (see Fig. 2, Table 3). Time spent sedentary and in moderate-to-vigorous activity during this period were negatively correlated (*r* = −0.552, *p* < 0.001, see Table 2).

A follow-up stepwise hierarchical regression analysis determined that the best model to predict long-term memory in the present dataset incorporated Sex, Age, Aerobic Fitness, time sedentary 0:56:20 to 1:51:20 following the first session, and time spent sedentary 0:50:40 up to the start of the second session (*R*^2^ = 0.32, *F* (5, 86) = 8.1, *p* < 0.001).

## Discussion

The aim of the present investigation was to examine the relationship between physical activity during the day and the maintenance of information in long-term memory. Findings revealed that neither the total amount of time spent sedentary nor time spent in moderate-to-vigorous physical activity during the day related to long-term memory. Such findings replicate those of Pontifex and colleagues^7^, who also failed to observe any relationship between long-term memory and time spent in moderate-to-vigorous physical activity as measured using accelerometry. However, it should be noted that Pontifex and colleagues^7^ utilized a 24-hour memory retention period which introduced sleep as a potential confound. Collectively then, these findings suggest that long-term memory is unaffected by the amount of physical activity or sedentary time, generally distributed over the course of the day.

Novel to the present investigation, however, was the determination of critical periods when physical activity engagement was more strongly associated with long-term memory in order to provide insight into the time-course of the effects of bouts of physical activity on long-term memory. Using an exploratory approach, two critical periods were identified as relating to the maintenance of information within long-term memory. The period occurring approximately 1 hour prior to the memory assessment was observed to be associated with both sedentary behavior and moderate-to-vigorous physical activity, with sedentary behavior negatively associated and physical activity positively associated with memory performance. It should be noted however, that these critical periods for sedentary behavior and moderate-to-vigorous physical activity were not perfectly aligned nor perfectly correlated (*r* = −0.55). Conceptually, such findings are not altogether unsurprising as it may be that a brief recovery period following moderate-to-vigorous physical activity is necessary prior to the retrieval of information from long-term memory for benefits to be observed; whereas lesser-intensities of physical activities can be engaged in right up to the start of the memory assessment. Interestingly, this period within one hour prior to the memory assessment replicates the relative timing used by much of the acute-exercise and cognition literature^33^,^34^, which has assessed aspects of attention and high-level cognitive processes immediately following engaging in a bout of exercise. Thus, the present investigation adds to this body of literature by investigating long-term memory processes over the entire span between encoding and retrieval, suggesting that avoiding sedentary behavior and engaging in physical activity during the period 1 hour prior to memory retrieval is associated with greater maintenance of information in long-term memory. The exact mechanisms underlying such a relationship are as of yet unknown. However, speculatively this modulation in long-term memory due to physical activity may reflect increased availability of norepinephrine following physical activity engagement. In support of this assertion, prior research in non-human animal models suggests that exercise may increase the availability of norepinephrine within the locus coeruleus, amygdala, and hippocampus^35^, which serves to increase the responsivity of the synaptic functions of cortical neurons in these regions^36^. As suggested by Murchison and colleagues^37^, this increased availability of norepinephrine within the hippocampus serves to alter aspects of information processing to aid in memory retrieval. Thus, it may be that engaging in physical activity during this critical period increases norepinephrine availability in the hippocampus, resulting in a greater ability to retrieve information from long-term memory. Given the findings of the present investigation, further research is necessary to better understand how these modulations in long-term memory associated with physical activity engagement immediately prior to memory retrieval relate to changes in hippocampal norepinephrine.

The other critical period when physical activity engagement was identified as being related to long-term memory was approximately 1 to 2 hours following the encoding of information into long-term memory. In contrast to views that suggest that any time is a good time to exercise, spending more time physically active during this critical period was associated with poorer maintenance of information within long-term memory. This suggests that engaging in physical activity during the period following memory encoding may impede the consolidation of information into long-term memory. Although speculative, one mechanism that may underlie such a relationship is retroactive interference. It is widely established that a memory trace can be disrupted if new information is acquired shortly after initial encoding. That is, long-term declarative memory is disrupted when multiple pieces of information are encoded and this effect is typically stronger when the information is semantically or phonologically similar^20^. Although it was previously believed that interference only affected information within the same memory system (e.g. declarative information only affected declarative memory and procedural information only affected procedural memory), new research has emerged to suggest that interference can also occur between different memory systems, as a result of competition for neural resources (Robertson, 2012). Thus, it may be that participating in physical activities requires activation of additional memory processes which create interference for the items recently placed within long-term memory. Physical activity during this period approximately 1 to 2 hours following the encoding of information may be especially problematic as memory is particularly vulnerable to interference shortly after acquisition and during memory reactivation^20^. As physical activity may serve to facilitate memory reactivation and retrieval processes, physical activity during this period may create a situation in which items recently stored within long-term memory are particularly vulnerable to interference in the memory consolidation process. Further research is clearly necessary however, to first replicate the association between physical activity during this critical period and the maintenance of information in long-term memory; and then to further investigate how motor control processes involved in physical activity participation may create interference for declarative memory.

## Conclusions

Collectively, findings from the present investigation suggest that in terms of long-term memory, the timing of the physical activity is of greater importance than the total proportion of time spent being physically active during the day. Engaging in physical activity during the critical window approximately 1 to 2 hours following the encoding of information was observed to be detrimental to the maintenance of information in long-term memory. However, physical activity during the period 1 hour prior to memory retrieval was associated with greater maintenance of information. Furthermore, these critical physical activity periods were each observed to be independently associated with long-term memory, even after controlling for the influences of aerobic fitness and demographic factors; with the combined model accounting for over 30% of the total observed variance. A limitation of the present investigation, however, is the reliance on cross-sectional empirical designs to reduce the potential for interference-related effects^20^. Thus, future investigations utilizing assessments of long-term memory that may be less prone to interference-related effects are necessary in order to better assess the effects of behavioral actions during the day on long-term memory using repeated-measures, within-subjects designs. Given the exploratory nature of the present investigation aimed at determining these critical periods when physical activity relates to long-term memory, further research also is necessary to validate the existence and relative timings of these critical periods. In particular, the data-driven approach to define these critical periods relies upon variation in activity levels across participants throughout the day. As the participant population was drawn from a college-aged young adult sample, the relative timing of these critical periods may have been influenced by greater homogeneity in sedentary behavior and physical activity participation at particular time points in the day inherent to classroom and campus related activities. Indeed, as evidence suggests that long-term memory is most sensitive to retroactive interference shortly after the initial encoding of information, the finding that physical activity during the 1 to 2 hour period following encoding was detrimental to long-term memory is somewhat odd. Thus, it may be that there was insufficient variability in physical activity participation immediately following the first session - as participants left the laboratory - for the data-driven analysis to identify this period. Future investigations are therefore necessary to specifically target these observed critical periods using a hypothesis based approach, rather than the exploratory approach utilized in the present investigation. It is also important to note that the mechanisms involved in these observed relationships may differ over the course of the lifespan, necessitating the need for future investigations to determine the relationship between physical activity during the day and memory consolidation in older adults and school-aged pediatric populations. Finally, beyond relying on cardiovascular intensity alone, our understanding of the relation between sedentary and physical activity behaviors during these critical periods to long-term memory would be further enhanced by the collection of more specific information regarding the particular activities that participants engage in. As it may be that the particular physical activities are of greater importance than the relative cardiovascular intensity. However, these findings provide initial evidence, from which future investigations can build upon, to suggest that long-term memory may be enhanced by more carefully attending to the relative timing of physical activity incorporated during the day. It would seem that superior long-term memory of declarative information occurs when physical activity opportunities are more strategically aligned to either fall prior to memory encoding — consistent with the existent literature base^21^,^23^,^24^,^25^, or in the period prior to memory retrieval.

## Additional Information

**How to cite this article**: Pontifex, M. B. *et al*. The Association between Physical Activity During the Day and Long-Term Memory Stability. *Sci. Rep.* **6**, 38148; doi: 10.1038/srep38148 (2016).

**Publisher's note:** Springer Nature remains neutral with regard to jurisdictional claims in published maps and institutional affiliations.

## Figures and Tables

**Table 1:** Participant demographic, fitness, and physical activity characteristics (±SD).

Measure	All Participants
N	92 (40 females)
Age (yrs)	19.1 ± 1.3 (min–max: 18–24)
Hispanic	2.2%
Non-White	13%
Body Mass Index (kg/m^2^)	23.8 ± 3.4 (min–max: 16.3–39.9)
VO_2_max (ml/kg/min)	43.1 ± 10.1 (min–max: 14.3–70.8)
VO_2_max Percentile	50.8 ± 35.9 (min–max: 3–97)
Percent Time Sedentary (<30% HRR)	85.13 ± 9.2 (min–max: 50.0–98.5)
Percent Time Light Physical Activity (30 to 40% HRR)	7.8 ± 4.2 (min–max: 1.3–25.4)
Percent Time Moderate Physical Activity (40 to 60% HRR)	5.8 ± 4.9 (min–max: 0.3–23.5)
Percent Time Vigorous Physical Activity (>60% HRR)	1.8 ± 2.8 (min–max: 0–15.2)
Minutes of Sedentary Behavior 0:56:20 to 1:51:20 Post-Encoding	50.0 ± 10.2 (min–max: 9.0–55.3)
Minutes of Sedentary Behavior 0:50:40 to 0:0:00 Pre-Retrieval	38.3 ± 10.9 (min–max: 1.3–51.0)
Minutes of Moderate-to-Vigorous Physical Activity 0:58:40 to 0:20:00 Pre-Retrieval	3.0 ± 7.0 (min–max: 0–36.7)
Proportion of items maintained between sessions (%)	91.0 ± 6.6 (min–max: 71.74–100.0)

Note: VO_2_max Percentile – based on normative values for VO_2_max (Shvartz & Reibold, 1990).

**Table 2:** Bivariate correlations between demographic factors, fitness, and physical activity with long term memory retention.

Variable	1.	2.	3.	4.	5.	6.	7.	8.	9.	10.
1. Long-Term Memory	—									
2. Age	−0.168	—								
3. Sex (0 = Female, 1 = Male)	−0.249*	0.030	—							
4. Race (0 = white, 1 = nonwhite)	−0.048	0.016	0.022	—						
5. Body Mass Index	−0.180	0.031	0.236**	−0.035	—					
6. Aerobic Fitness (VO_2_max Percentile)	0.211*	0.026	0.294***	−0.214*	−0.105	—				
7. Percent Time Sedentary (<30% HRR)	−0.058	−0.093	0.026	−0.023	0.087	0.046	—			
8. Percent Time Moderate-to-Vigorous Physical Activity (≥40% HRR)	0.120	0.045	−0.065	0.098	−0.062	−0.038	−0.915***	—		
9. Bouts of Sedentary Behavior 0:56:20 to 1:51:20 Post-Encoding	0.205*	0.042	−0.009	−0.159	0.034	0.126	0.033	−0.067	—	
10. Bouts of Sedentary Behavior 0:50:40 to 0:0:00 Pre-Retrieval	−0.309**	0.167	−0.004	0.018	0.154	−0.039	0.391***	−0.399***	−0.168	—
11. Bouts of Moderate-to-Vigorous Physical Activity 0:58:40 to 0:20:00 Pre-Retrieval	0.210**	−0.020	−0.080	0.110	−0.070	0.010	−0.358***	0.357***	0.064	−0.552***

Note: *p ≤ 0.05, **p ≤ 0.025, ***p ≤ 0.001.

**Table 3:** Summary of the final step of the hierarchical regression analyses for the relationship between fitness and physical activity with long-term memory retention.

Variable	*R*^2^	*R*^2^ Change	*F* Change	B	B SE	β	*t*	*p*
Aerobic Fitness	0.16	0.05*	4.8	0.04	0.02	0.225	2.2	0.03
Percent Time Sedentary	0.18	0.02	1.8	−0.09	0.07	−0.130	1.3	0.19
Percent Time Moderate-to-Vigorous Physical Activity	0.17	0.01	1.5	0.12	0.10	0.119	1.2	0.23
Bouts of Sedentary Behavior								
0:56:20 to 1:51:20 Post-Encoding	0.27	0.11***	12.5	0.21	0.06	0.330	3.5	<0.001
0:50:40 to 0:0:00 Pre-Retrieval	0.24	0.08**	8.5	−0.17	0.06	−0.280	2.9	0.004
Bouts of Moderate-to-Vigorous Physical Activity								
0:58:40 to 0:20:00 Pre-Retrieval	0.22	0.06**	6.6	0.24	0.09	0.253	2.6	0.01

Note: *p ≤ 0.05, **p ≤ 0.025, ***p ≤ 0.001. Model for Cardiorespiratory Fitness included Age and Sex. All models for physical activity included Age, Sex, and Cardiorespiratory Fitness.
